# Proof of principle: Preoperative cognitive reserve and brain integrity predicts intra-individual variability in processed EEG (Bispectral Index Monitor) during general anesthesia

**DOI:** 10.1371/journal.pone.0216209

**Published:** 2019-05-23

**Authors:** Carlos Hernaiz Alonso, Jared J. Tanner, Margaret E. Wiggins, Preeti Sinha, Hari K. Parvataneni, Mingzhou Ding, Christoph N. Seubert, Mark J. Rice, Cynthia W. Garvan, Catherine C. Price

**Affiliations:** 1 Department of Clinical and Health Psychology, University of Florida College of Public Health and Health Professions, Gainesville, Florida, United States of America; 2 Department of Orthopedic Surgery, University of Florida College of Medicine; Gainesville, Florida, United States of America; 3 Department of Biomedical Engineering, University of Florida Herbert Wertheim College of Engineering, Gainesville, Florida, United States of America; 4 Department of Anesthesiology, University of Florida, Gainesville, Florida, United States of America; 5 Department of Anesthesiology, Vanderbilt University Medical Center, Nashville, Tennessee, United States of America; Nathan S Kline Institute, UNITED STATES

## Abstract

**Background:**

Preoperative cognitive reserve and brain integrity may explain commonly observed intraoperative fluctuations seen on a standard anesthesia depth monitor used ubiquitously in operating rooms throughout the nation. Neurophysiological variability indicates compromised regulation and organization of neural networks. Based on theories of neuronal integrity changes that accompany aging, we assessed the relative contribution of: 1) premorbid cognitive reserve, 2) current brain integrity (gray and white matter markers of neurodegenerative disease), and 3) current cognition (specifically domains of processing speed/working memory, episodic memory, and motor function) on intraoperative neurophysiological variability as measured from a common intraoperative tool, the Bispectral Index Monitor (BIS).

**Methods:**

This sub-study included participants from a parent study of non-demented older adults electing unilateral Total Knee Arthroplasty (TKA) with the same surgeon and anesthesia protocol, who also completed a preoperative neuropsychological assessment and preoperative 3T brain magnetic resonance imaging scan. Left frontal two-channel derived EEG via the BIS was acquired preoperatively (un-medicated and awake) and continuously intraoperatively with time from tourniquet up to tourniquet down. Data analyses used correlation and regression modeling.

**Results:**

Fifty-four participants met inclusion criteria for the sub-study. The mean (SD) age was 69.5 (7.4) years, 54% were male, 89% were white, and the mean (SD) American Society of Anesthesiologists score was 2.76 (0.47). We confirmed that brain integrity positively and significantly associated with each of the cognitive domains of interest. EEG intra-individual variability (squared deviation from the mean BIS value between tourniquet up and down) was significantly correlated with cognitive reserve (r = -.40, p = .003), brain integrity (r = -.37, p = .007), and a domain of processing speed/working memory (termed cognitive efficiency; r = -.31, p = .021). Hierarchical regression models that sequentially included age, propofol bolus dose, cognitive reserve, brain integrity, and cognitive efficiency found that intraoperative propofol bolus dose (p = .001), premorbid cognitive reserve (p = .008), and current brain integrity (p = .004) explained a significant portion of intraoperative intra-individual variability from the BIS monitor.

**Conclusions:**

Older adults with higher premorbid reserve and less brain disease were more stable intraoperatively on a depth of anesthesia monitor. Researchers need to replicate findings within larger cohorts and other surgery types.

## Introduction

Anesthesiology research suggests that depth of sedation as measured by two-channel derived EEG monitors is a risk factor for negative postoperative outcomes such as delirium and mortality particularly in older adults [1]. One such monitor is the Bispectral Index Monitor (BIS) [2]. This monitor converts electroencephalograph (EEG) readings from the frontal cortex into a simplified number for rapid assessment of hypnotic depth. The value of the simplified number is debated in the literature [3]. For example, rapid fluctuations can occur in EEG derived BIS values for the same patient throughout surgery even during stable periods of anesthesia monitoring. For this reason, we know that some anesthesiologists consider derived EEG metrics unreliable and therefore disregard the readings. Previous research, however, demonstrates the potential clinical utility of derived EEG variability [4]. We propose that anesthesiology providers should expect variability, particularly in older adults with varying degrees of cognitive reserve and possible neurodegenerative pathologies. We base our theoretical rationale on biological and behavioral research demonstrating that intra-individual variability occurs and is a marker of neuronal instability.

Neurophysiological intra-individual variability indicates compromised regulation and organization of neural networks [5] with increased pathology theoretically altering the clarity of neural signal-to-noise [6]. Increased variability is more pronounced among individuals with low psychometrically defined intelligence [7], schizophrenia [8], Alzheimer’s disease, and diffuse Lewy body disease [9]. Neurophysiological variability associates with brain shrinkage, cortical thinning, and frontoparietal atrophy [10–12]. Intra-individual variability increases with white matter pathology [13, 14] and reduction in specific gray matter regions such as the entorhinal cortex [15]. For these reasons, intra-individual variability is a construct that may provide insight into intraoperative anesthesia brain response.

This study’s working hypothesis is preoperative measures of cognition and brain integrity in older adults predict intraoperative neurophysiological variability during anesthesia. We applied a theoretical framework of cognitive reserve, brain integrity (gray and white matter markers of neurodegenerative disease), and current cognition (specifically processing speed/working memory, episodic memory). First, premorbid cognitive reserve represents psychosocial, experiential factors (e.g., greater educational attainment), and genetic factors (e.g., childhood intelligence) that enable the brain to withstand injury [16]. Cognitive reserve represents foundational synaptic density distinct from neurodegenerative brain pathologies [16–18]; individuals with significant amounts of reserve can have substantial neurodegenerative disease markers without clinical manifestation. Second, current brain integrity represents the status of an individual’s gray and white matter as seen on brain magnetic resonance (MR). Entorhinal cortical thickness, leukoaraiosis (i.e., white matter disease), and lateral ventricular volume are three well-established neuroimaging markers of early neurodegenerative pathology. Entorhinal cortices connect the temporal neocortices with the hippocampi; entorhinal thickness decreases with age [19] and more so for individuals with prodromal or diagnosed Alzheimer’s disease [20]. Leukoaraiosis (LA) [21] associates with small vessel cerebrovascular disease [22]. Ventricular volume correspondingly enlarges with brain tissue loss [23]. Finally, manifesting from individuals’ premorbid reserve and the amount of current brain integrity is the third component of our model: current cognition [17]. Cognitive domains of the frontal-subcortical/ thalamocortical system (i.e., processing speed, working memory; heretofore called “cognitive efficiency”) and medial/lateral temporal systems (i.e., episodic memory encoding and retrieval) are most vulnerable to aging vascular pathologies.

To assess our hypotheses, we first confirmed expected relationships between cognitive reserve, current brain integrity, and current cognition (i.e., cognitive efficiency, episodic memory). Secondly, we examined relationships between preoperative cognitive and brain integrity variables with EEG intra-individual variability during preoperative awake and intraoperative time periods. We then conducted hierarchical analyses to assess how each theoretical composite explained intraoperative EEG intra-individual variability. The regression models sequentially included age and propofol bolus dose, cognitive reserve, brain integrity, and cognitive efficiency. The order of variables included in the sequence of models was determined theoretically according to hypothesized effects on EEG intra-individual variability.

## Methods

### Participants

The present investigation was performed as a secondary study using data collected through a larger parent study approved by the University of Florida’s Institutional Review Board-01 (IRB #487–2012). Written informed consent was obtained from all participants and the original study was registered prior to participant enrollment at clinicaltrials.gov (NCT01786577, Principal investigator: Price, Date of registration: 01/07/2013). The authors followed principles from the Declaration of Helsinki. Neuroimaging data from a subset of the participants are reported elsewhere in a peer-reviewed published manuscript [24].

### Recruitment

Participants were recruited between August 2013 and March 2016. One surgeon (HKP) approached eligible individuals undergoing primary total knee replacement surgery to consider participation in this voluntary, federally funded research investigation conducted through the University of Florida. If interested in the study, participants completed a written informed consent form followed by a cognitive telephone screener [25] and a comprehensive history and systems interview to confirm inclusion and exclusion criteria. Qualifying participants then completed an in-person rating of comorbidity [26], depressive symptom severity [27], activities of daily living [28], preoperative neuropsychological testing, and a preoperative brain magnetic resonance imaging scan.

#### Inclusion criteria for parent study

All participants had to meet the following inclusion criteria: aged 60 or older, English as the primary language, have osteoarthritis or comparable joint pain, have intact activities of daily living and have baseline neuropsychological testing unsupportive for dementia criteria per Diagnostic and Statistical Manual of Mental Disorders, Fifth Edition. Two neuropsychologists (CCP, JJT) reviewed the baseline data to confirm test scores met the expected ranges for non-demented individuals. Participants also had to have a complete set of BIS recordings, preoperative brain MRI sequences, and preoperative neuropsychological measures of interest.

#### Exclusion criteria for parent study

Individuals were excluded if they had a history of head trauma, documented learning or seizure disorder, less than a sixth-grade education, substance abuse in the last year, major cardiac disease, or chronic medical illness known to induce encephalopathy (e.g., liver disease). Additional exclusion criteria included: deviation from anesthetic protocol, inability to tolerate normal dose of hypnotic agent during anesthetic induction, and incomplete record of electronic perioperative data collection.

#### Additional criteria for current sub-study

For the sub-study, additional requirements were complete EEG data collection intraoperatively and complete T1 MRI acquisition. The final sub-study sample included 54 participants with complete preoperative neuropsychological and brain measures and complete intraoperative BIS recording.

### Surgical and anesthetic protocols

Adequate anesthesia is the balance between the amount of medication the central nervous system is exposed to and the level of surgical stimulation. Therefore, an arousal response can be triggered by a significant change in surgical stimulation. We designed our study to focus on a phase of the operation where the influence of nociceptive input would be minimal.

Protocols were standardized, with surgery participants receiving intravenous midazolam (1–4 mg) followed by continuous femoral nerve block (CFNB) and single-injection subgluteal sciatic nerve block with 20 mL and 30 mL, respectively, of 0.5% ropivacaine as a bolus injection. The CFNB was continued with ropivacaine 0.2% at an infusion rate of 10 mL per hour. No opioids were added. Propofol, fentanyl, and rocuronium were used for anesthesia induction and intubation. Patients were ventilated with an air oxygen mixture to maintain an end tidal carbon dioxide at 35 ± 5 mm, FiO_2_ between 0.5 and 0.7; anesthesia was maintained with inhaled sevoflurane and intravenous fentanyl and rocuronium. Propofol boluses were administered as needed to maintain desirable target BIS range between 40 and 60. Total knee replacement surgery was done in a standard manner for all patients by the same surgeon. A tourniquet was used for all cases set to 250 mm Hg and inflated prior to incision and deflated just prior to closure. Bony preparation was done by intramedullary instrumentation for the femoral side and extramedullary for the tibial side. The anterior and posterior cruciate ligaments were sacrificed for all patients and implants were fixed to the bone using bone cement. Perioperative information, including surgery events (e.g., induction, intubation, incision, tourniquet inflation and release, etc.), anesthetic drugs, and intraoperative medications, were recorded on a standardized study data collection sheet and confirmed with the official anesthesia record.

### Primary predictor and outcome variables of interest

#### Cognitive reserve, brain integrity, and current cognition

As part of a more extensive federally funded investigation, each participant completed a planned preoperative neuropsychological evaluation and brain magnetic resonance (MR) imaging scan. Preoperative brain and neuropsychological predictor variables of interest were standardized to the participant sample of interest. Although external normative values are available for the neuropsychological variables, there are no known external normative values for neuroanatomical variables. In order to maintain consistency in the standardization of brain and cognitive variables, we based the final brain and cognitive standardized scores on the participant sample.

**Cognitive reserve** is a concept to partially explain why there is not a direct relationship between clinical symptoms (e.g., memory) and factors (e.g., neurodegenerative pathology) that should affect function; it is not measured directly and is typically estimated from sociobehavioral proxies like education, IQ, and others [29]. We did not rely on participant reported years of education due to reservations that it does not capture quality of education and lifetime gained knowledge [16]. Rather, premorbid cognitive reserve was operationalized as a composite of two measures shown to be resistant to neurodegenerative disease processes and considered to be estimates of premorbid intelligence: 1) word reading ability from the Wide Range Achievement test (WRAT), and 2) Vocabulary subtest of the Wechsler Abbreviated Scale of Intelligence (WASI). For test descriptions please see [30]. These scores were standardized and averaged.

**Brain integrity** was *a priori* defined using three common measures of brain disease: entorhinal thickness, leukoaraiosis (LA) volume, and total lateral ventricular volume.

Left entorhinal thickness—The entorhinal cortex is an early marker of medial temporal disease pathology involved in Alzheimer’s disease and is a recipient of cholinergic input. We focused on the left entorhinal cortex due to the dominance of verbal measures in our sample and evidence of asymmetrical degeneration with neurodegenerative risk factors and disease [31]. T1 images were processed through the FreeSurfer Version 6.0 pipeline with measures calculated from the automatic subcortical segmentation and Desikan–Killiany–Tourville atlas cortical parcellation [32].

Frontal lobe leukoaraiosis—LA is common in aging and is associated with increased risk of dementia. LA within the frontal lobe hypothetically interferes with frontal cortical-subcortical connections important for a range of cognitive functions, including speeded processing [33]. A reliable rater measured all scans for LA using FLAIR sequences with in-house macros using previously published methods [34]. To calculate LA within the frontal lobes, we merged cortical parcellations into lobes and then used automatic algorithms (FreeSurfer’s *mri_aparc2aseg*) to segment white matter within each lobe. FLAIR images were then rigid body registered to the T1 images for each participant with these transformations then applied to the LA masks. The volume of LA within the frontal lobes was then calculated.

Ventricular volume—Larger lateral ventricular volume is a risk factor for dementia [35].

To acquire these three MR metrics, participants completed preoperative MRI within a Siemens 3T Verio scanner with an 8-channel head coil. We acquired T1-weighted (176 contiguous slices, one mm^3^ voxels, TR–TE = 2500–3.77 ms) and Fluid Attenuated Inversion Recovery (FLAIR; 176 contiguous slices, 1 mm^3^ voxels, TR–TE = 6000–395 ms) scans. As a control variable, total intracranial volume (TICV) was estimated by FreeSurfer *maskvol* algorithm [36].

To form the brain integrity composite we averaged the standardized scores from the left entorhinal thickness in mm, frontal LA volume in mm^3^ (controlled for TICV), and total lateral ventricle volume in mm^3^ (controlled for TICV). The direction of the z-scores was standardized such that positive scores indicated better brain integrity.

**Current cognition** represents preoperative cognitive abilities we hypothesized would relate with intraoperative EEG variability. We chose to examine cognitive domains vulnerable to aging and surgery with anesthesia [37, 38]. Cognitive domains and associated tests are described below. More in-depth test descriptions are available elsewhere [30].

Processing speed and working memory (heretofore called ‘Cognitive efficiency’ for simplicity)—are domains dependent on frontal-striatal circuitry and dorsolateral prefrontal to parietal activation [30, 39]. Processing speed and working memory are also elements of executive function that change with normal aging and neurodegenerative disorders [22, 40], and are known to change after major surgery [38]. Processing speed measures included: Digit Symbol subtest from the Wechsler Adult Intelligence Scale, 3^rd^ edition (WAIS-III; total symbols coded in 120 seconds); Stroop Color Word Test, Word subtest (total words read correctly in 45 seconds); and Trail Making Test, Part A (time in seconds). Working memory measures included: Digit Span Backward Span (WAIS-III; longest span backwards), Letter-Number Sequencing (WAIS-III; total score), and Spatial Span Backward from the Wechsler Memory Scale, 3^rd^ edition (WMS-III; total score). The direction of the working memory and processing speed z-scores was standardized such that positive scores indicated better performance. Working memory and processing speed scores were then averaged to create each individual’s final cognitive efficiency composite.

Episodic verbal memory—is a primary domain of memory altered by neurodegenerative diseases [30] and is also known to change after surgery [37, 38]. We assessed verbal memory using a 12 word list learning measure (Hopkins Verbal Learning Test-Revised (HVLT-R; delay, recognition)), and a paragraph story test (WMS-III, Logical Memory Test Delay). To create the Episodic Verbal Memory composite we used the total number of words recalled in delay (HVLT-R), the recognition discrimination index score (HVLT-R), and the total number of details recalled from paragraph stories (WMS-III). These scores were standardized and averaged for each individual’s final composite.

Motor Function—as measured by index finger tapping has been shown to be less susceptible to change after TKA [37]. It was included in this study as a dissociate variable to the primary cognitive domains of interest (i.e., cognitive efficiency, episodic memory). The motor function composite was based on mean number of index finger taps across separate 10-second trials of the dominant and non-dominant hands (Finger Tapping Test). These scores were standardized and averaged for each individual’s final composite.

### Derived EEG intra-individual variability

The Bispectral Index (BIS, Aspect Medical Systems, Newton, MA, USA) uses a dimensionless monotonic index to record anesthesia depth on a scale from 100 (awake state) to 0 (deep coma) [2]. The electrodes are integrated into a sensor that is placed on the left forehead. Monitors like the BIS were originally designed to help detect and prevent awareness and memory formation during the surgery process. The BIS algorithm initially processes the frontal EEG to detect the presence of cerebral suppression (i.e., burst suppression or persistent suppression) and performs a fast Fourier transform (FFT) on the waveform. Data from the FFT are used to compute the ratio of higher frequency waves (30 to 47 Hz) to other waves of lower frequency (11 to 20 Hz), and to compute the bispectrum, which measures the phase coupling between high frequency (40 to 47 Hz) and a broader frequency range (0.5 to 47 Hz) of EEG waves. Reliability and validity of the BIS are published [2]. With the exception of the bispectral analysis, these features can be qualitatively assessed from the raw EEG and nonproprietary processed parameters [41]. Corresponding to standard BIS placement, the sensors were placed on the left frontal region just above the eyebrow. BIS Index values were acquired on the same machine for each participant. The same smoothing rate, impedance checking, and filter was applied on the monitor settings for all participants. A BIS value was saved every minute as an average sum of two-second epochs over the previous minute. We collected BIS measures perioperatively: 1) baseline BIS measurements, acquired for 5 minutes preoperatively prior to receiving midazolam and alfentanil administration for the femoral nerve block; and 2) intraoperative BIS measurements, acquired throughout the surgery from pre-induction to time of waking. Intraoperative EEG intra-individual variability was examined from time of tourniquet inflation to release establishing a consistent period of examination while reducing extraneous variation that may result during induction and emergence. Baseline and intraoperative EEG intra-individual variability scores were calculated as the squared deviation from the mean BIS value over the duration of measurement.

### Control variables

The following control variables were considered in the analyses: age, Charlson Comorbidity Index (CCI) [26], American Society of Anesthesiologists score (ASA), body mass index, depressive symptom severity via the Geriatric Depression Scale (GDS) [27], intraoperative propofol bolus (mg), and total intracranial volume.

### Statistical analyses

Data were checked for implausible values, missingness, and distributional form. Cognitive composites and brain integrity scores were deemed normally distributed using graphical displays (e.g., Q-Q plots). Preoperative and intraoperative intra-individual variability values were found skewed on visual inspection. Therefore, the EEG derived variability values were log transformed. Pearson product moment correlations examined predictions regarding two-channel intraoperative EEG intra-individual variability, cognitive reserve, brain integrity, and cognitive domains. Hierarchical regression analyses were conducted to assess how each theoretical composite explained intraoperative EEG intra-individual variability. The regression models sequentially included age and propofol bolus dose, cognitive reserve, brain integrity, and cognitive efficiency. The order of variables included in the sequence of models was determined theoretically according to hypothesized effects on EEG intra-individual variability. All analyses were performed using SPSS version 24. The level of significance was set at .05.

## Results

Fifty-four participants met inclusion criteria for the sub-study. The mean (SD) age was 69.5 (7.4), 54% were male, 89% were white, and the mean (SD) American Society of Anesthesiologists score was 2.76 (0.47). Two neuropsychologists reviewed the cognitive and behavioral data. On a measure of instrumental and basic activities of daily living [28], participants were independent for physical self-maintenance and for telephone use, medication management, and finances. One participant was semi-independent at managing medications and shopping. See Table 1 for participant additional demographics, cognitive, and brain imaging metrics. See Table 2 for derived EEG and intraoperative variables. Within the final sub-study sample we identified 12 participants who received propofol boluses during time of tourniquet up to tourniquet down; 8 participants received one bolus, 3 participants received two boluses, and 1 participant received four boluses. See supplementary S1 and S2 Tables for group differences (no propofol bolus n = 42 vs propofol bolus n = 12) between demographic, cognitive, and brain imaging metrics and intraoperative variables. Bolus groups differed by race and intraoperative EEG derived variance.

**Table 1:** Descriptive statistics for demographics, cognitive, and brain variables of interest (n = 54).

Variables	Mean ± standard deviation or % (n)	Minimum, maximum
**Demographics**		
Age (years)	69.52 ± 7.35	60, 85
ASA^1^		---
1	2% (1)	
2	20% (11)	
3	78% (42)	
Body Mass Index	32.35 ± 5.59	23.40, 43.19
CCI^2^	0.46 ± 0.82	0, 4
Education (years)	15.15 ± 2.59	10, 21
Sex		---
Female	46% (25)	
Male	54% (29)	
GDS^3^	4.26 ± 4.56	0, 24
Race		---
White	89% (48)	
Non-White	11% (6)	
Activities of Daily Living	29.17 ± 1.32	23, 30
**Cognitive Reserve Raw Scores**		
Vocabulary	59.70 ± 6.98	47, 71
WRAT^4^	51.48 ± 3.88	41, 57
**Brain Integrity Raw Components (mm**^**3**^)		
Entorhinal thickness (mm)	3.20 ± 0.26	2.65, 3.72
Frontal leukoaraiosis	2399.67 ± 3553.49	15.00, 23158.00
Total intracranial volume	1559619.15 ± 158578.17	1292485.00, 1888908.00
Ventricular volume	33486.29 ± 18090.23	9452.60, 92396.70
**Preoperative Cognitive–Memory Raw Scores by Domain**		
Processing speed^5^		
Digit symbol	57.41 ± 12.95	29.00, 88.00
Stroop color word test; word subtest	88.91 ± 9.56	69.00, 100.00
Trail making test part A	37.76 ± 14.45	19.00, 95.00
Working memory^5^		
Digits span backwards	4.93 ± 1.34	2.00, 7.00
Letter number sequencing	9.52 ± 2.56	4.00, 15.00
Spatial span backwards	6.94 ± 1.86	2.00, 11.00
Episodic Memory		
HVLT-R^6^ delay	7.87 ± 2.80	1.00, 12.00
HVLT-R recognition	10.35 ± 1.26	6.00, 12.00
LM^7^ delay	26.50 ± 7.46	8.00, 41.00
Motor function		
Finger tapping- (dominant hand)	44.81 ± 7.96	25.30, 64.20
Finger tapping- (non-dominant hand)	40.71 ± 6.91	25.80, 58.80

^1^ASA = American Society of Anesthesiologists Physical Status Classification System;

^2^CCI = Charlson Comorbidity Index;

^3^GDS = Geriatric Depression Scale;

^4^Wide Range Achievement test;

^5^Processing speed and working memory were combined into a theoretical composite termed “cognitive efficiency”;

^6^HVLT-R = Hopkins Verbal Learning Test-Revised;

^7^LM = Wechsler Memory Scale-Third Edition Logical Memory Delay subtest.

Note: Z- scores based on participant sample. This established consistency across all measures (i.e. neuropsychological and neuroanatomical). Z-scores for neuropsychological measures from published normative references do not change the result of the findings or interpretation.

**Table 2:** Descriptive statistics for derived EEG intra-individual variance and intraoperative variables (n = 54).

Variables	Mean ± standard deviation	Minimum, Maximum
Total propofol dose (mg)^1^	208.52 ± 95.67	100.00, 750.00
Fentanyl dose (mcg)^2^	109.72 ± 60.54	0.00, 225.00
Tourniquet time (minutes)^3^	78.98 ± 10.52	59.00, 104.00
Preoperative variance^4^	5.87 ± 10.13	0.14, 54.97
Intraoperative variance^5^	47.60 ± 55.02	3.91, 279.43

^1^Total Propofol dose = total propofol dose administered intraoperatively;

^2^Fentanyl dose = total fentanyl dose administered intraoperatively;

^3^Tourniquet time = minutes from tourniquet inflation to tourniquet release;

^4^Preoperative variance = mean derived frontal EEG intra-individual variability for five minutes following proper two-channel lead placement and signal stabilization during full consciousness and prior to nerve block placement;

^5^Intraoperative variance = mean derived frontal EEG intra-individual variability calculated over the time from tourniquet inflation to release.

### Correlations among cognitive reserve, brain integrity, and current cognition

**Fig 1:**
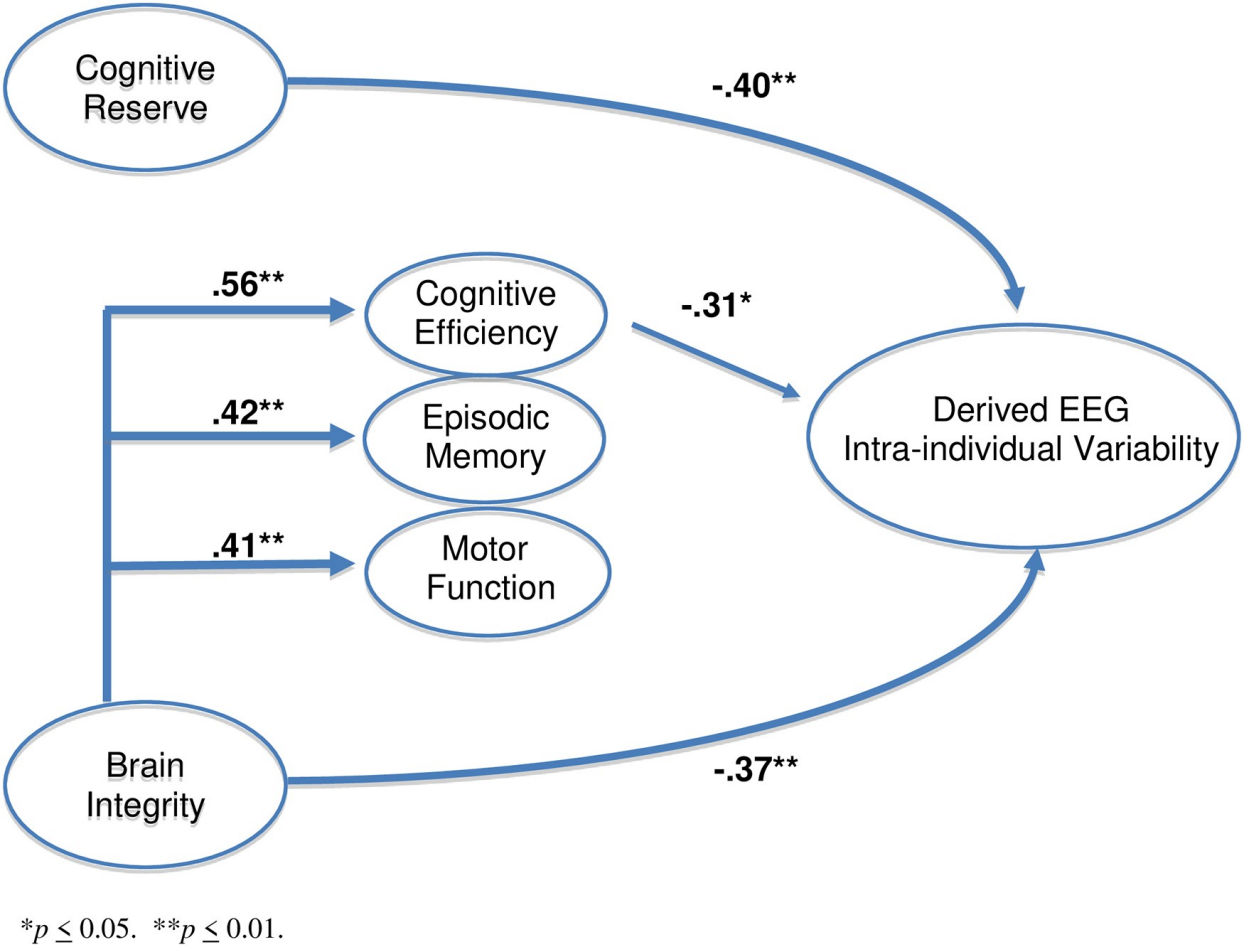
Correlation coefficients between each predictor variable and derived EEG intra-individual variability.

Working memory and processing speed were strongly correlated (r = .64, p < .001), suggesting appropriateness of combining them into a single composite. Current brain integrity positively associated with each current cognition domain, cognitive efficiency (r = .56, p < .0001), episodic memory (r = .42, p = .001), and motor function (r = .41, p = .002), but not cognitive reserve (r = .24, p = .071). Cognitive reserve positively associated with cognitive efficiency (r = .55, p < .001) and episodic memory (r = .30, p = .029). Fig 1 shows the theoretical model depicting relationships among components of cognitive reserve, brain integrity, and current cognition (cognitive efficiency, episodic memory, motor function) relative to derived EEG intra-individual variability from time of tourniquet inflation to tourniquet release.

### Associations between predictor variables of interest and BIS derived intra-individual variability

Baseline (preoperative) derived two-channel EEG intra-individual variability were not found to be associated with premorbid cognitive reserve (r = .08, p = .573), current brain integrity (r = -.08, p = .612), cognitive efficiency (r = -.01, p = .991), episodic memory (r = .02, p = .886), or motor function (r = .18, p = .233). Preoperative (baseline) variability was not found to be associated with intraoperative variability (r = -.08, p = .591).

**Fig 2:**
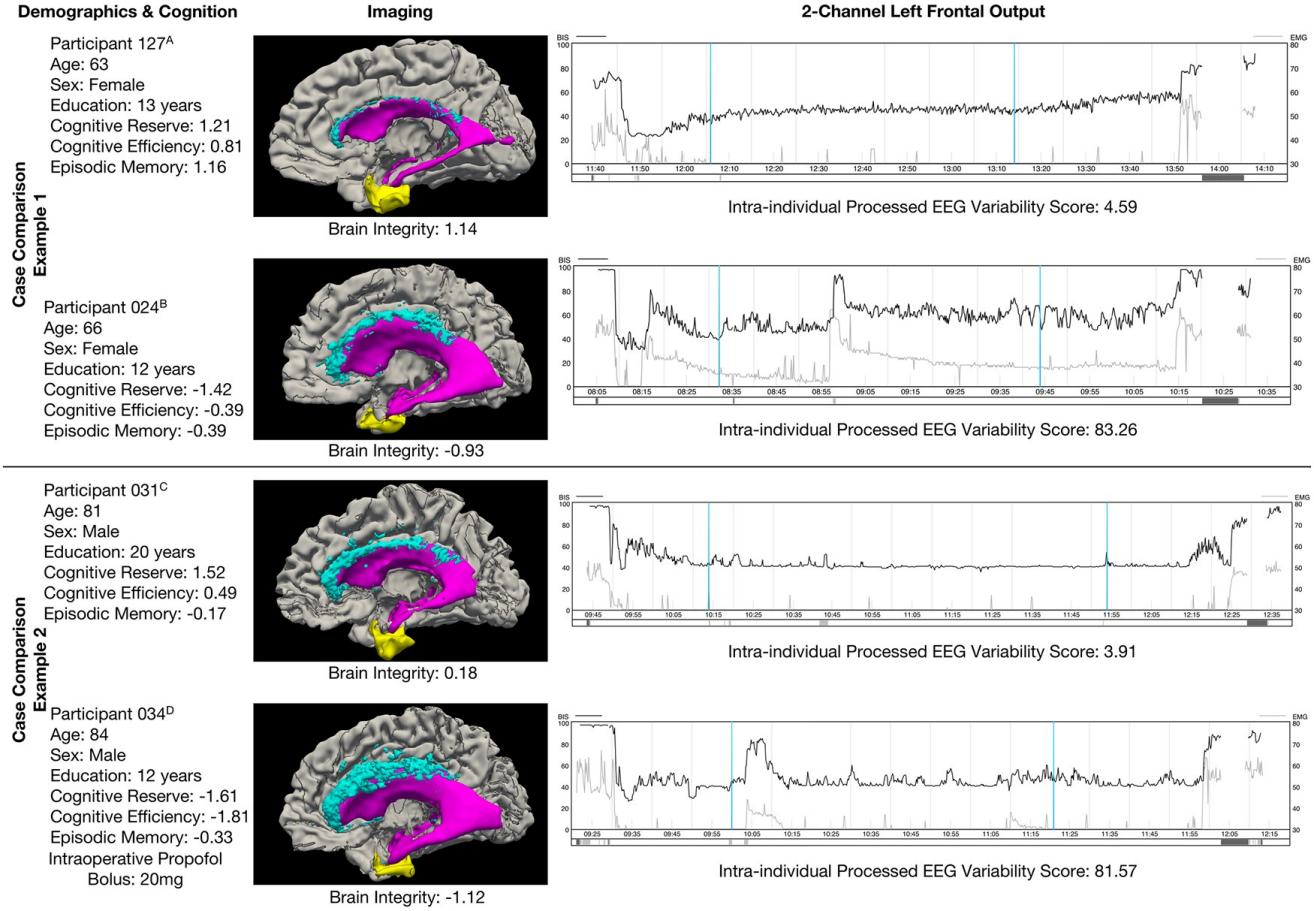
Case examples with predictor variables and 2-channel EEG output. The top two rows (case comparison example 1) presents two individuals in their 60’s electing TKA. The bottom two rows (case comparison example 2) presents two individuals in their 80’s electing TKA. The left column presents case demographics and final standardized scores for primary predictor variables of interest. The middle column depicts the brain variables of interest (entorhinal thickness in yellow; leukoaraiosis in teal; ventricle size in pink) and standardized brain integrity score. The right column shows the 2-channel EEG output and processed/derived EEG intra-individual variability score. Blue vertical lines indicate time of tourniquet inflation (left line) and tourniquet release (right line).

Intraoperative derived EEG intra-individual variability was significantly associated with premorbid cognitive reserve (r = -.40, p = .003) and current brain integrity (r = -.37, p = .007). Of the current cognitive domains, only cognitive efficiency predicted intraoperative derived EEG intra-individual variability (r = -.31, p = .021; episodic memory, r = .01, p = .912; motor, r = -.17, p = .223). Fig 2 includes representative case comparisons depicting the relationship between predictor variables of interest and 2-channel EEG output.

### Hierarchical regression model results

After adjusting for age and intraoperative propofol bolus, we found that cognitive reserve and brain integrity explained a significant amount of intraoperative derived EEG intra-individual variability. Results of all regression models are displayed in Table 3.

**Table 3:** Summary of hierarchical regression analysis for variables predicting intraoperative BIS variance (n = 54).

	Model 1			Model 2			Model 3			Model 4		
Variable	B^1^	SE B^2^	β^3^	B	SE B	β	B	SE B	β	B	SE B	β
Age	0.004	0.018	0.032	0.005	0.016	0.033	-0.024	0.018	-0.172	-0.021	0.019	-0.150
Propofol bolus*	0.012	0.004	0.392**	0.012	0.004	0.396**	0.012	0.003	0.385**	0.013	0.004	0.413**
Cognitive reserve				-0.457	0.134	-0.400**	-0.348	0.132	-0.304*	-0.435	0.157	-0.380*
Brain integrity							-0.687	0.244	-0.382**	-0.800	0.268	-0.445**
Cognitive efficiency										0.232	0.227	0.165
*R*^*2*^		0.153			0.313			0.408			0.421	
*F* for change in *R*^*2*^		4.615*			11.620**			7.904**			1.041	

^1^B- Regression coefficient

^2^SE B- Standard error

^3^ β- Standardized regression coefficient

*p ≤ 0.05.

**p ≤ 0.01.

Age was not a significant predictor (p = 0.267) but propofol bolus dose (mg) from tourniquet up to down explained 15% of the variance. The overall model with cognitive reserve, brain integrity, and cognitive efficiency was significant (F = 6.978, p < 0.001) with cognitive reserve (Beta = -0.38, p = .008) and brain integrity (Beta = -0.45, p = .004) being the only additional significant predictors. Cognitive efficiency was not significant in this model (Beta = 0.17, p = .313). This model explained 42.1% of the variance in BIS variability.

## Discussion

Our data show that intra-individual variability observed on the derived output of a patient’s BIS monitor does not solely reflect noise. Rather, rapid fluctuations particularly during a stable period of the operation where the influence of nociceptive input is minimal appears to reflect participants’ premorbid cognitive reserve or current level of brain pathology. Within our sample of non-demented older adults, individuals with higher cognitive reserve and less brain pathology showed less intraoperative intra-individual variability from time of tourniquet up to tourniquet down. Combined with intraoperative propofol dose, metrics of premorbid cognitive reserve and preoperative brain integrity explained 41% of the intraoperative BIS variance. Preoperative scores on tests of processing speed and working memory (cognitive efficiency) also associated with a more stable EEG pattern, but this association did not significantly explain additional variance over cognitive reserve and brain integrity in the regression model. These findings correspond to previous reports that intra-individual variability is a marker of central nervous system integrity, with increased intra-individual variability even on a derived BIS metric providing meaningful information about patients’ brain status. We hypothesize the pattern of individual variance indicates dysfunctional modulation of select neurotransmitters and frontal cortex–mediated processes [5].

Cognitive reserve and brain integrity together helped to explain a large portion of variance in our patients’ BIS response. Cognitive reserve explained the most amount of observed variance (16%) and brain integrity explained an additional 9%. These findings fit theory; premorbid intellectual ability is the foundation upon which disease burden accumulates, and depending on the strength of the foundation and burden load, clinical signs manifest [17]. Epidemiological evidence suggests cognitive reserve begins in childhood and accumulates throughout life [16]. In contrast, common neurodegenerative disorders (Alzheimer’s disease, small vessel vascular disease, Parkinson’s disease) begin in the middle to older age years. For example, the entorhinal cortex, known for its rich cholinergic network [42], reaches peak at age 44 and then declines in thickness more rapidly if there is Alzheimer’s disease pathology [43, 44]. Cerebrovascular disease also increases with age, eventually disrupting cortico-striatal-thalamic circuitry, thalamic gaiting, processing speed, working memory, and inhibition [33]. Although complementary, cognitive reserve and brain integrity designate two separate variables worthy of consideration when attempting to analyze BIS output.

Intra-individual variability was also partially explained by measures of current preoperative frontal-striatal functioning. Only the domain of processing speed/working memory (cognitive efficiency) predicted BIS variability; episodic memory and motor function composites had flat and weak associations, respectively. These findings underscore the importance of cortico-striatal-thalamic circuitry in anesthesia response for non-demented older adults electing TKA. Similar assertions regarding the role of fronto-striatal circuits have been suggested by Giattino and colleagues [45] who showed that a preoperative metric of current cognition (a composite of verbal memory, abstraction and visuospatial orientation, visual memory, attention and concentration) positively associated with intraoperative alpha power in a sample of 15 older adults studied via 32-channel EEG and 35 older adults undergoing a variety of non-cardiac or non-neurological surgical procedures after surgical incision.

We acknowledge study limitations. First, the study has a small sample size. However, despite the small sample size, the results show the relevance of baseline cognitive reserve, MRI quantified brain integrity, and preoperative cognition for anesthesiology consideration. The investigative questions we present are worth further investigation with more sophisticated EEG technology, and replication with a larger sample and additional surgical populations. Second, we identified 12 participants who had an additional unexpected propofol bolus during time of tourniquet up to tourniquet down. Including intraoperative propofol bolus dose in our hierarchical regression did not alter the expected pattern of results. A comparison of demographics for bolus/no bolus participants was somewhat surprising; results identified a difference in race by group such that in the bolus group 33% of the participants were African American compared to 5% of the non-bolus group. Future studies need to examine patient diversity status and BIS output. Third, despite best efforts we were not able to acquire raw EEG BIS data for more fine grained analyses addressing power frequencies and anteriorization. This is an area for future study. Researchers need to investigate how reserve, brain integrity, and neurophysiological intraoperative intra-individual variability relate to the concept of anteriorization and alpha power. Finally, we did not include other measurements of autonomic dysfunction in our investigation. We encourage future investigators to consider measures of autonomic dysfunction in their research on the BIS, for autonomic dysfunction is associated with dementia [46] and autonomic dysfunction associates with processed intraoperative EEG [4, 47].

Despite study weaknesses there are numerous study strengths. Our results demonstrate that derived BIS output may provide valuable information about patient brain integrity. Design strengths include a consistent anesthetic protocol and use of the same surgeon for all patients. In efforts to examine a more refined stability of neural networks under general anesthesia, our protocol refrained from using medications and anesthetics known to alter EEG signals (i.e. ketamine, nitrous oxide). Additionally, we chose a stable time period during the surgical procedure that reduced intra-individual variability associated with anesthesia induction and emergence. Cognitive reserve was derived from robust measures of reading and vocabulary rather than education alone. We approach brain integrity as a composite of common brain disease markers that indicate early neurodegenerative pathology. These markers had clinical relevance even in non-demented older adults. Finally, we had *a priori* theories that cognitive efficiency and memory would be clinically relevant to intraoperative intra-individual variability. We used a motor function metric to dissociate frontal lobe behaviors. This metric demonstrated derived BIS measurement is largely explained by frontal-subcortical/frontal-parietal elements of executive function and not premotor and primary motor cortex variables alone. Our findings underscore the importance of preoperative brain and cognitive status on intraoperative EEG as measured by commercial devices commonly used in the operating room setting. These findings are relevant given the rate of undiagnosed cognitive impairment in the community and preoperative settings [48–50], and given that TKA surgeries have doubled from 2000 to 2010 and continue to increase [51].

Overall, study findings reiterate that intra-individual EEG variability has hypothetical relevance to understanding anesthesia response in older adults. Intra-individual associations to cognitive reserve and brain integrity were observed only during times of surgical and anesthesia exposure. It remains unknown how intraoperative BIS EEG intra-individual variability predicts clinical outcome. We are addressing this question in an ongoing investigation.

## Supporting information

S1 Table Group (no propofol bolus n = 42 vs propofol bolus n = 12) differences for demographics, cognitive, and brain variables of interest. ^1^ASA = American Society of Anesthesiologists Physical Status Classification System; ^2^CCI = Charlson Comorbidity Index; ^3^GDS = Geriatric Depression Scale; ^4^Wide Range Achievement test; ^5^Processing speed and working memory were combined into a theoretical composite termed “cognitive efficiency”; ^6^HVLT-R = Hopkins Verbal Learning Test-Revised; ^7^LM = Wechsler Memory Scale-Third Edition Logical Memory Delay subtest. Note: Z- scores based on participant sample. This established consistency across all measures (i.e. neuropsychological and neuroanatomical). Z-scores for neuropsychological measures from published normative references do not change the result of the findings or interpretation.
(PDF)

S2 Table Group (no propofol bolus n = 42 vs propofol bolus n = 12) differences for derived EEG and intraoperative variables. ^1^Bolus frequency = number of boluses administered from tourniquet inflation to tourniquet release; ^2^Intraoperative propofol = total propofol dose administered from tourniquet inflation to tourniquet release (bolus); ^3^Total propofol dose = total propofol dose administered intraoperatively; ^4^Fentanyl dose = total fentanyl dose administered intraoperatively; ^5^Tourniquet time = minutes from tourniquet inflation to release; ^6^Preoperative variance = mean derived frontal EEG intra-individual variability for five minutes following proper two-channel lead placement and signal stabilization during full consciousness and prior to nerve block placement; ^7^Intraoperative variance = mean derived frontal EEG intra-individual variability calculated over the time from tourniquet inflation to release.
(PDF)
