# Supplementary material for: Proof of principle: Preoperative cognitive reserve and brain integrity predicts intra-individual variability in processed EEG (Bispectral Index Monitor) during general anesthesia
Source: PLoS One. 2019 May 23;14(5):e0216209. doi: 10.1371/journal.pone.0216209 (PMC6532861; doi:10.1371/journal.pone.0216209)
Supplement: S1 Table — 1ASA = American Society of Anesthesiologists Physical Status Classification System; 2CCI = Charlson Comorbidity Index; 3GDS = Geriatric Depression Scale; 4Wide Range Achievement test; 5Processing speed and working memory were combined into a theoretical composite termed “cognitive efficiency”; 6HVLT-R = Hopkins Verbal Learning Test-Revised; 7LM = Wechsler Memory Scale-Third Edition Logical Memory Delay subtest. Note: Z- scores based on participant sample. This established consistency across all measures (i.e. neuropsychological and neuroanatomical). Z-scores for neuropsychological measures from published normative references do not change the result of the findings or interpretation. (PDF) [file pone.0216209.s001.pdf]

Variables	No Bolus (n=42)	Bolus (n=12)	
	**Mean ± standard deviation or %(n)**	**Mean ± standard deviation or %(n)**	**p value**
**Demographics**			
Age (years)	69.64 ± 7.00	69.08 ± 8.81	.842
ASA[1] (1:2:3 only)	1:9:32	0:2:10	.798
1	2% (2)	0% (0)	---
2	21% (9)	17% (2)	---
3	76% (32)	83% (10)	---
Body Mass Index	32.86 ± 5.77	30.52 ± 4.66	.161
CCI[2]	0.33 ± 0.65	0.92 ± 1.16	.914
Education (years)	15.17 ± 2.72	15.08 ± 2.19	.431
Sex			
Female	50% (21)	33% (4)	---
Male	50% (21)	67% (8)	---
GDS[3]	4.05 ± 3.94	5.00 ± 6.47	.529
Race			
White	95% (40)	67% (8)	
Non-White	5% (2)	33% (4)	<.01
**Cognitive Reserve Raw Scores**			
Vocabulary	60.05 ± 7.07	58.50 ± 6.83	.501
WRAT[4]	51.64 ± 3.58	50.92 ± 4.94	.642
**Brain Integrity Raw Scores (millimeter cubed; mm^3)**			
Entorhinal thickness (mm)	3.19 ± 0.26	3.21 ± 0.25	.797
Frontal leukoaraiosis	2276.79 ± 3659.10	2829.75 ± 3267.53	.621
Total intracranial volume	1535768.62 ± 137614.42	1643096.00± 201997.69	.106
Ventricular volume	33232.70 ± 16102.71	34373.81± 24682.07	.882
**Preoperative Cognitive–Memory Raw Scores by Domain**			
Processing speed[5]			
Digit symbol	58.35 ± 12.71	54.08 ± 13.79	.349
Stroop color word; word subtest	90.00 ± 9.26	85.08 ± 10.02	.146
Trail making test part A	36.60 ± 12.22	41.83 ± 20.65	.417
Working memory[5]			
Digits span backwards	4.93 ± 1.31	4.92 ± 1.51	.980
Letter number sequencing	9.67 ± 2.50	9.00 ± 2.80	.467
Spatial span backwards	7.21 ± 1.82	6.00 ± 1.76	.051
Episodic memory			
HVLT-R[6] delay	8.14 ± 2.79	6.91 ± 2.75	.191
HVLT-R recognition	10.35 ± 1.38	10.33 ± 0.78	.490

LM[7] delay	26.98 ± 7.66	24.83 ± 6.78	.360
Motor function			
Finger tapping- (dominant hand)	44.49 ± 6.67	45.90 ± 11.73	.697
Finger tapping- (non-dominant hand)	39.84 ± 6.86	43.78 ± 6.43	.081
