# Supplementary material for: Proof of principle: Preoperative cognitive reserve and brain integrity predicts intra-individual variability in processed EEG (Bispectral Index Monitor) during general anesthesia
Source: PLoS One. 2019 May 23;14(5):e0216209. doi: 10.1371/journal.pone.0216209 (PMC6532861; doi:10.1371/journal.pone.0216209)
Supplement: S2 Table — 1Bolus frequency = number of boluses administered from tourniquet inflation to tourniquet release; 2Intraoperative propofol = total propofol dose administered from tourniquet inflation to tourniquet release (bolus); 3Total propofol dose = total propofol dose administered intraoperatively; 4Fentanyl dose = total fentanyl dose administered intraoperatively; 5Tourniquet time = minutes from tourniquet inflation to release; 6Preoperative variance = mean derived frontal EEG intra-individual variability for five minutes following proper two-channel lead placement and signal stabilization during full consciousness and prior to nerve block placement; 7Intraoperative variance = mean derived frontal EEG intra-individual variability calculated over the time from tourniquet inflation to release. (PDF) [file pone.0216209.s002.pdf]

**Variables**	**No Bolus (n=42)**	**Bolus (n=12)**	**p-value**
	**Mean ± standard deviation or % (n)**	**Mean ± standard deviation or % (n)**	
Bolus frequency[1]			
1	---	67% (8)	---
2	---	25% (3)	---
4	---	8% (1)	---
Intraoperative propofol (mg)[2]	---	54.17 ± 52.48	---
Total propofol dose (mg)[3]	189.76 ± 51.34	274.17 ± 168.01	.112
Fentanyl dose (mcg)[4]	112.50 ± 62.29	100.00 ± 55.39	.670
Tourniquet time (minutes)[5]	78.57 ± 10.33	80.41 ± 11.52	.624
Preoperative variance[6]	5.90 ± 10.80	5.71 ± 7.11	.947
Intraoperative variance[7]	34.82 ± 40.50	92.33 ± 75.28	.025
